# Streamwater responses to reduced nitrogen deposition at four small upland catchments in Norway

**DOI:** 10.1007/s13280-020-01347-3

**Published:** 2020-06-13

**Authors:** Øyvind Kaste, Kari Austnes, Heleen A. de Wit

**Affiliations:** 1 Norwegian Institute for Water Research, Gaustadalleen 21, 0348 Oslo, Norway; 2 Centre for Coastal Research, University of Agder, PO box 422, 4604 Kristiansand, Norway

**Keywords:** Atmospheric deposition, Catchments, Long-term trends, Nitrogen leaching, Surface waters

## Abstract

Reduced emissions of nitrogen (N) in Europe have resulted in decreasing atmospheric deposition since 1990. Long-term data (1988–2017) from four small Norwegian catchments located along gradients in N deposition, rainfall, and organic carbon (C) show different responses to 25–30% reductions in N deposition during the same period. At three sites the decreased N deposition caused reduced leaching of nitrate to surface water, whereas the westernmost site showed no decrease, probably due to thin soils with low C:N ratio, poor vegetation cover and high precipitation. The loss of total N to streamwater constituted 30–50% of the N deposition. Losses via denitrification are unknown but assumed to be low, as a major fraction of the catchments are well-drained. Hence, the study sites seem to continue to accumulate N, presumably mostly in soil organic matter. Although atmospheric N deposition has declined, ambient loads might still exceed long-term sustainable levels in these vulnerable ecosystems.

## Introduction

Emissions of sulphur oxides (SOx), nitrogen oxides (NOx) and reduced nitrogen (NHx) in Europe have been reduced by approximately 90, 55 and 20%, respectively, since 1990 (EEA 2017). The reduction in sulphur (S) deposition has resulted in a clear improvement in the acidification status of acid-sensitive lakes and streams, with increasing acid neutralizing capacity (ANC) and improved conditions for fish and other freshwater organisms (Garmo et al. 2014). Reduced deposition of sulphate (SO_4_) and sea-salts has also resulted in an increase in dissolved organic carbon (DOC) in surface waters (Monteith et al. 2007), a proxy for dissolved organic matter (DOM) which is a vector for transport of organic N.

Nitrogen can contribute to acidification in the same way as sulphur if it leaks out of the soil as nitrate (NO_3_). During the early 1990s, before the atmospheric deposition of N started to decline, there was a concern that the excess N from atmospheric sources would lead to N saturation and massive leakage of NO_3_ and acidifying components from soil to water (Aber et al. 1989; Stoddard 1994). Many natural or semi-natural terrestrial ecosystems, especially in central Europe and eastern North America, showed signs of N saturation with increased leaching of NO_3_ (Wright et al. 2001; Kopacek et al. 2005). In some cases, drought incidents and insect attacks worsened the situation and led to forest decline and massive NO_3_ leaching (Oulehle et al. 2013; Seidl et al. 2017). In Fennoscandia there were reports of increasing N concentrations in upland surface waters during the 1980s and 1990s (Ågren 1983; Lepistö 1995; Kaste et al. 1997). Many of these ecosystems are particularly sensitive to acidification. They have thin soils and sparse vegetation and a limited capacity to retain N from atmospheric deposition.

There is no consistent relationship between the general decline in deposition of N in Europe over the last 30 years and the leaching losses of inorganic and organic N from natural or semi-natural catchments in the same regions (Garmo et al. 2014; Vuorenmaa et al. 2018). Results from national monitoring programmes differ, with some sites showing reduced concentrations of inorganic N (Kopacek et al. 2005; Vuorenmaa et al. 2018) while other sites show increasing trends (Rogora et al. 2013). It is still not clear which factors are causing catchments to respond differently to reduced N input from atmospheric sources. One possible explanation could be that N deposition has not been sufficiently reduced to produce significant downward trends. In addition, the increase in DOM is expected to be associated with an increase in organic N, which counteracts the effect of declining N deposition on the leaching of total nitrogen (TN).

Climate change may also have affected N transport from land to water. Increased air and soil temperatures lead to increased mineralisation, longer growing season and increased vegetation growth. More plant biomass not only results in increased uptake of nutrients but also more litterfall and larger stocks of organic matter (OM) in the soils that may leach to surface waters (Finstad et al. 2016). Higher summer temperatures can also lead to forest decline due to drought and increased risk of insect attacks (Seidl et al. 2017). With increasing rainfall OM accumulated in the soils is more susceptible to leaching from soils to adjacent surface waters (de Wit et al. 2016).

Here, we quantify long-term changes in deposition and leaching of inorganic and organic N in four small, upland catchments in Norway. These cover the east–west gradient in N deposition, rainfall and DOC. The goal is to (i) describe the N leaching pattern after the N deposition started its decline around 2000, (ii) identify factors that may explain why the catchments respond differently to reduced N deposition, and (iii) assess how different catchment characteristics and climate factors might influence the catchment’s ability to retain N from long-range transported air pollution.

## Materials and methods

### Study catchments

**Fig. 1:**
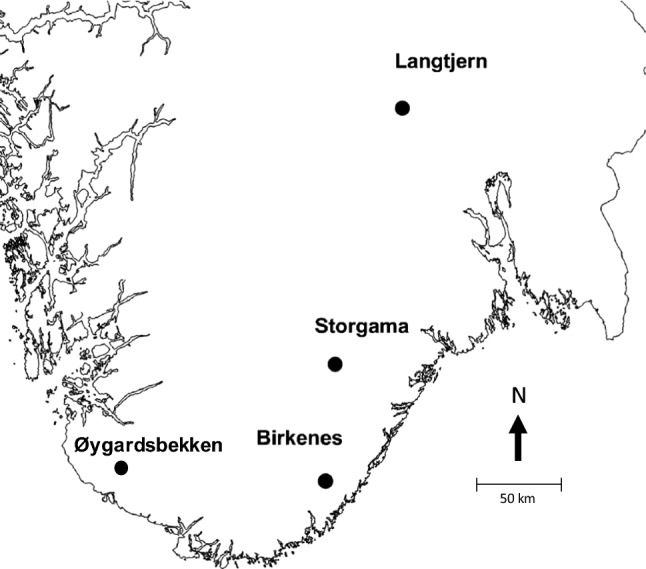
Map of southernmost Norway showing the four study sites

The four long-term monitoring sites selected for this study (Fig. 1) are part of the Norwegian monitoring programme on effects of long-range transported air pollution (Johannessen 1995). They are small, semi-natural catchments (0.4–4.8 km^2^) located along distinct gradients in N deposition, climate and land cover (Table 1). The catchments were selected because of their extensive and long-term data on atmospheric deposition, stream flow, and chemistry of soils and surface water—and that they all have atmospheric deposition as the only external N source (N fixation assumed negligible). Langtjern, Storgama, and Birkenes have been monitored continuously since the early 1970s; Øygardsbekken since 1993.

**Table 1:** Site characteristics. From Garmo and Skancke (2019) and www.nevina.nve.no. Temperature and precipitation data represent the 30-year normal, 1961–1990, calculated by the Norwegian Water Resources and Energy Directorate (NVE)

	Langtjern	Storgama	Birkenes	Øygardsbekken
Catchment size (km^2^)	4.8	0.6	0.41	2.6
Elevation (masl)	510–750	580–690	200–300	185–544
Latitude (Deg N)	60.372	59.052	58.386	58.622
Longitude (Deg E)	9.727	8.654	8.244	6.107
Productive forest (%)	5	11	90	4
Marginal land^a^ (%)	74	59	3	83
Peatland (%)	16	22	7	6
Lakes (%)	5	8	–	7
Bedrock	Gneiss	Granite	Granite, biotite	Gneiss, anorthosite
Ann. mean temp. (°C)	1.2	2.9	5.9	4.8
Ann. mean precip. (mm year^−1^)	791	1037	1405	2063

^a^Langtjern and Birkenes: Low-productive forest, Storgama and Øygardsbekken: Exposed bedrock or scattered forest/shrubs on shallow soils

Langtjern, the easternmost catchment, is situated at 500–700 m elevation and has a typical inland climate with relatively cold winters and warm summers. The mineral soils have developed on glacial till on felsic gneisses and granites. Deeper peaty soils are common close to streams and small lakes, in addition to poorly drained depressions. The catchment is dominated by unproductive forest of Norway spruce (*Picea abies*), Scots pine (*Pinus sylvestris*), and birch (*Betula pubescens*). The Lake Langtjern, located at the bottom of the catchment has a surface area of 0.23 km^2^.

Storgama is also at 600–700 m above sea level. It is situated 60 km from the south coast and has relatively cold winters and warm summers. Mineral soils have developed in a shallow layer of glacial till on granitic bedrock. The main mineral soil type is podzol. Shallow peaty deposits have developed on poorly drained sites in the catchment. The vegetation is predominantly a sparse unproductive forest of Scots pine (*Pinus sylvestris*) and some birch (*Betula pubescens*) with undergrowth of heather. Storgama has larger areas with exposed bedrock than any of the other catchments. The weir at the bottom of the catchment is at the outlet of a small pond.

Birkenes is further south and closer to the coast, at an altitude of 200–300 m. The climate here is milder, with rainy winters and often hot summers. The site is substantially influenced by seasalt deposition, which also affects streamwater chemistry. Soils are acid brown earths and podzols on granitic bedrock. Peaty deposits have developed on poorly drained sites in the catchment. On the slopes, well-drained thin organic layers on gravel or bedrock are common. The catchment is dominated by productive forest, consisting of 135-year old Norway spruce (*Picea abies*) (Kvaalen et al. 2002) with some Scots pine (*Pinus sylvestris*) and birch (*Betula pubescens*). There are no lakes in the catchment.

Øygardsbekken lies farthest to the southwest, at about 200–500 m altitude. The site is close to the North Sea and has a typical coastal climate with high precipitation amounts in all seasons, mild winters and relatively cool summers. Øygardsbekken is located in the part of Norway with the highest deposition of long-range transported air pollution (Tørseth and Semb 1997). The bedrock geology consists of migmatites, banded gneisses and anorthosite covered by glacial sediments. Soils are thin and patchy, especially in the upper parts of the catchment where also exposed bedrock commonly occurs. The vegetation is largely heathland shrubs, with grasses, moors and scattered birch forest. Approximately, 7% of the catchment is covered by small lakes.

For comparison with regional N trends we have also included long-term data (1990–2017) from the outlet of four large rivers in which the study catchments are located; River Drammenselva (catchment area 17 034 km^2^, including Langtjern), River Nidelva (4015 km^2^, incl. Storgama), River Tovdalselva (1855 km^2^, incl. Birkenes), and River Bjerkreimselva (705 km^2^, incl. Øygardsbekken). All river basins are part of the Norwegian River Monitoring programme (Kaste et al. 2018), and their catchments are dominated by mountains or forested areas (range 90–98%) with only small factions of agricultural land (1–4%). For further comparison with regional N trends, we have also included long-term data (1988–2017) for 40 upland lakes located along the same geographical gradient and sampled each autumn as part of the Norwegian monitoring programme on effects of long-range transported air pollution (Johannessen 1995). All lakes have atmospheric deposition as the only external N source.

### Water chemistry and hydrology

Chemical samples have been collected weekly (fortnightly at Øygardsbekken before 2010) and analysed unfiltered at NIVA for several parameters, of which pH, calcium (Ca), sulphate (SO_4_), chloride (Cl) nitrate (NO_3_), ammonium (NH_4_), total nitrogen (TN), and total organic carbon (TOC) are presented in this paper. All variables have been monitored during the entire period, except NH_4_, which was analysed from 2000 at Øygardsbekken, from 2004 at Storgama and from 2005 at Langtjern and Birkenes. pH is analysed by potentiometry; NO_3_, SO_4_ and Cl, Ca and NH_4_ by ion chromatography, TN by automated colorimetry (after oxidation with peroxodisulphate), and TOC by spectophotometry after UV oxidation. Total organic nitrogen (TON) was calculated as TN minus NO_3_ and NH_4_. For years with lacking NH_4_ data, TON is calculated with estimated NH_4_ concentrations (mean of the five first years with measured NH_4_ data). Given that NH_4_ represent a minor fraction of TN in the study catchments (< 5%) it represents only a small source of error in the TON estimates. Level of detection (LOD)/level of quantification (LOQ) for the analyses are: Ca (0.0007/0.002 mg L^−1^), Cl (0.0017/0.005 mg L^−1^), SO_4_ (0.0017/0.005 mg L^−1^), NO_3_ (0.3/1 µg N L^−1^), NH_4_ (0.07/2 µg N L^−1^), TN (3.3/10 µg L^−1^), and TOC (0.03/0.1 mg L^−1^). The estimated uncertainties of the analyses are < 20%. All samples have been analysed at the same laboratory during the whole monitoring period, and minor changes in analytical methods and detection limits ensure comparable data over time.

Water discharge is recorded continuously at V-notch weirs at the outlets of each catchment. The gauging stations at Langtjern, Storgama and Birkenes are administered and operated by the Norwegian Water Resources and Energy Directorate (NVE). The gauging station at Øygardsbekken was operated by NIVA until 2003. Thereafter, water discharge has been scaled from the NVE station 27.26.0 Hetland (69.7 km^2^) which has its headwaters 5 km west from Øygardsbekken. There was a 10-year overlapping period between the gauging stations before 2003, and a data comparison show that the short-term flow dynamics at Øygardsbekken were captured relatively good with scaled data from the NVE station Hetland, with an R^2^ of 0.56 between measured and estimated daily flow during the period 1993–2002.

Element fluxes were calculated for each day using measured water discharges at each sampling site multiplied by the solute concentration interpolated from the weekly samples. The daily calculated data were then aggregated to annual fluxes of each element. Annual flow-weighted concentrations were calculated from yearly fluxes divided by the annual water discharge.

### Atmospheric deposition

The atmospheric N deposition data are obtained from the nearest monitoring stations operated by the Norwegian Institute for Air Research (NILU) as part of the Norwegian programme for monitoring of long-range transported air pollution (Aas et al. 2019). The nearest deposition stations at Langtjern, Storgama, Birkenes and Øygardsbekken are Brekkebygda, Treungen, Birkenes and Vikedal, respectively. Precipitation chemistry and precipitation amounts are determined by bulk sampling on a daily or weekly basis. Calculation of wet N deposition from the bulk deposition measurements is done by weighting daily or weekly concentrations of NO_3_ and NH_4_ by the corresponding precipitation volume relative to the total annual precipitation volume. More details related to analytical methods, quality control and flux calculations are described elsewhere (Aas et al. 2019).

Except for Øygardsbekken, the deposition stations are near the study catchments (< 7 km). At Øygardsbekken, the closest operational deposition station (Vikedal) is located 94 km to the north. To check the representativity of this station for the Øygardsbekken catchment, we have compared N deposition time series at Vikedal with two local deposition stations that were operated during the 1990s; Ualand (15 km to south-east) and Skreådalen (50 km to north-east). During an eight-year period between 1992 and 1999 when all three stations were in operation, the deviations in wet N deposition were small (7 and 15% higher at Vikedal compared to Ualand and Skreådalen, respectively). Tørseth and Semb (1997) found that annual precipitation at Øygardsbekken during 1993–1995 was 8–24% (mean 18%) higher than at Ualand, and the annual deposition of total inorganic nitrogen (TIN; NO_3_ + NH_4_) was 12–23% (mean 19%) higher than at Ualand. Based on this cross-comparison, deposition data from Vikedal are regarded as representative for the Øygardsbekken catchment.

### Trend analyses

The Mann–Kendall test (Hirsch and Slack 1984) was used to evaluate temporal trends in atmospheric deposition, water discharge and element fluxes. To minimize the risk of autocorrelation the analyses were performed on annual means only. The MK test is robust against outliers, missing data, and does not require normal distribution of data. The method was used to determine monotonic trends based on the values of the test statistic (*Z* score). Slopes were calculated using the Sen estimator (Sen 1968), which is the median of the slopes calculated from all pairs of values in the data series. This slope estimate is little affected by data outliers and missing data. Trends were considered as statistically significant at the 5% level.

## Results

### Water quality status, 2013–2017

#### Element concentrations in streams

All four catchments are acid-sensitive with low calcium concentrations (< 1 mg L^−1^) (Table 2). They span a relatively large gradient in TOC, with the highest concentrations in the east (Langtjern: 11.2 mg L^−1^) and gradually decreasing concentrations towards west (Øygardsbekken: 1.8 mg L^−1^). Langtjern and Storgama are little affected by sea-salts (mean Cl concentrations 0.4–0.9 mg L^−1^), while Birkenes and Øygardsbekken are more affected with average Cl concentrations of 4.4–7.4 mg L^−1^, respectively.

**Table 2:** Runoff chemistry, 2013–2017. Mean concentrations (± standard deviation), element ratios, and annual fluxes. Asterisk denotes the non-marine fraction which is calculated by subtracting the marine contribution estimated from the ratio of SO_4_ to Cl in seawater

	Langtjern	Storgama	Birkenes	Øygardsbekken
Concentrations				
pH	5.1 ±0.2	5.0 ±0.2	4.9 ±0.2	5.5 ±0.3
Ca (mg L^−1^)	0.8 ±0.2	0.4 ±0.1	0.6 ±0.2	0.5 ±0.1
SO_4_* (mg L^−1^)	0.7 ±0.2	0.6 ±0.3	1.3 ±0.4	0.6 ±0.4
Cl (mg L^−1^)	0.4 ±0.1	0.9 ±0.5	4.4 ±1.0	7.4 ±2.4
NO_3_ (µg N L^−1^)	11 ±10	28 ±40	97 ±56	150 ±73
NH_4_ (µg N L^−1^)	9 ±6	14 ±14	12 ±12	6 ±18
TON (µg L^−1^)	249 ±39	243 ±60	199 ±69	103 ±33
TN (µg L^−1^)	268 ±38	285 ±57	307 ±72	259 ±76
TOC (mg L^−1^)	11.2 ±1.9	6.5 ±1.5	6.7 ±2.5	1.8 ±0.8
Element ratios				
NO_3_:TN (g g^−1^)	0.05	0.12	0.34	0.56
TON:TN (g g^−1^)	0.92	0.82	0.63	0.41
TOC:TON (g g^−1^)	45	28	35	18
Fluxes				
Water flow (mm year^−1^)	754	1127	1314	2213
NO_3_ (kg N km^−2^ year^−1^)	9	39	138	318
NH_4_ (kg N km^−2^ year^−1^)	6	16	15	14
TON (kg km^−2^ year^−1^)	179	260	256	232
TN (kg km^−2^ year^−1^)	194	316	409	564
TOC (kg km^−2^ year^−1^)	8161	7228	9003	4200

Although the deposition of S and N has declined significantly since the 1980s and 1990s, the four catchments are still strongly acidified. Concentrations of non-marine SO_4_ are highest at Birkenes (1.3 mg L^−1^) and somewhat lower in the other three catchments (0.6-0.7 mg L^−1^). Birkenes also has the lowest pH (4.9 on average), while Øygardsbekken had highest pH with 5.5 on average during 2013–2017.

Even though concentrations of TN are roughly the same at all sites (average: 259–307 µg L^−1^), there are large differences in the mix of N fractions. The overall picture is that inorganic N (mainly NO_3_) is lowest in the NE and increases towards the SW. The concentrations of NH_4_ are usually low at all stations. The regional pattern in organic N is opposite to NO_3_ and follows the regional distribution of TOC, with the highest concentrations in the NE and decreasing concentrations towards the SW.

#### Element fluxes in streams

There is a strong precipitation gradient in southern Norway with lowest rainfall in the east and increasing towards the west. This is also evident at the four study sites, with annual water flow at Øygardsbekken about three times higher than at Langtjern (Table 2). This makes the regional gradient in NO_3_ even greater if looking at fluxes rather than concentrations; the NO_3_ flux at Øygardsbekken was 35 times higher than at Langtjern during 2013–2017. Regarding total organic nitrogen (TON), the regional differences are dampened by higher concentrations in the east being offset by increasing runoff in the west.

There are also strong regional gradients in the ratio of different fractions of N to C. The NO_3_:TN ratio increases from 0.05 at Langtjern to 0.56 in Øygardsbekken, while the TON:TN ratio shows the opposite pattern. It is also interesting to note that the TOC:TON ratio decreases from east (Langtjern: 45) to west (Øygardsbekken: 18).

### Long-term trends, 1988–2017

#### Atmospheric N deposition

**Fig. 2:**
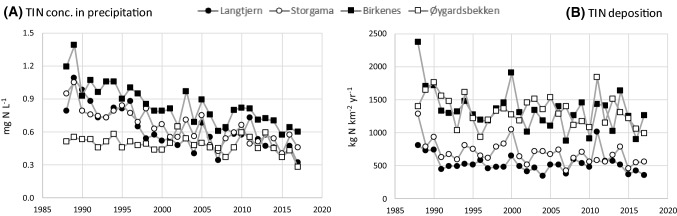
Atmospheric deposition of inorganic nitrogen (TIN); **a** Annual volume-weighted TIN concentrations in precipitation, **b** annual wet TIN deposition. Data from precipitation stations operated by the Norwegian Institute for Air Research (NILU): Brekkebygda (near Langtjern), Treungen (near Storgama), Birkenes, and Vikedal (near Øygardsbekken)

The concentrations of NO_3_ in precipitation have shown a significant decrease at all stations since 1988 (Fig. 2a, Table 3). For NH_4_, there has been a decline at all stations except at Øygardsbekken (NILU station Vikedal).

**Table 3:** Trends 1988^a^–2017 in (a) atmospheric deposition, (b) flow-weighted stream concentrations (c) stream fluxes, (d) element ratios in streams, and (e) stream fluxes vs. atmospheric inputs. Significance levels are indicated by *(*p *< 0.05) and **(*p *< 0.01), non-significant trends by ‘n.s.’. Upward and downward trends are indicated by Sen slope estimates

	Langtjern	Storgama	Birkenes	Øygardsbekken
(a) Atmospheric deposition				
*Concentrations in precipitation*				
NO_3_ (mg N L^−1^ year^−1^)	− 0.008**	− 0.008**	− 0.009**	− 0.003**
NH_4_ (mg N L^−1^ year^−1^)	− 0.009**	− 0.006**	− 0.008**	− 0.0, n.s.
Precipitation (mm year^−1^)	+ 15.6**	+ 10.0*	+ 20.6*	+ 4.3, n.s.
*Wet deposition:*				
NO_3_ (kg N km^−2^ year^−1^)	− 2.0, n.s.	− 5.8**	− 8.1*	− 10.2**
NH_4_ (kg N km^−2^ year^−1^)	− 2.6, n.s.	− 3.3*	− 5.8, n.s.	− 0.2, n.s.
TIN (kg km^−2^ year^−1^)	− 4.3, n.s.	− 8.8**	− 13.9*	− 10.8*
(b) FW stream concentrations				
NO_3_ (mg N L^−1^ year^−1^)	− 0.69**	− 4.60**	− 1.78*	− 1.30, n.s.
TON (mg L^−1^ year^−1^)	+ 0.82, n.s.	+1.31*	+1.49*	+ 0.73, n.s.
TN (mg L^−1^ year^−1^)	+ 0.07, n.s.	− 3.42**	− 0.00, n.s.	− 1.40, n.s.
TOC (mg L^−1^ year^−1^)	+ 0.10**	+ 0.08**	+ 0.08**	+ 0.03**
(c) Stream fluxes				
Water discharge (mm year^−1^)	+ 9.7**	+ 6.7, n.s.	+ 5.1, n.s.	+ 32.3**
NO_3_ (kg N km^−2^ year^−1^)	− 0.27**	− 3.87**	− 1.17, n.s.	+ 1.5, n.s.
TON (kg km^−2^ year^−1^)	+ 2.49**	+ 2.54*	+ 2.56, n.s.	+ 3.10*
TN (kg km^−2^ year^−1^)	+ 2.31**	− 1.42, n.s.	+ 0.91, n.s	+ 3.29, n.s.
TOC (kg km^−2^ year^−1^)	+ 158**	+ 129**	+ 143**	+ 111**
(d) Element ratios in streams				
NO_3_:TN (kg N kg^−1^ year^−1^)	− 0.003**	− 0.012**	− 0.005**	− 0.030, n.s.
TON:TN (kg kg^−1^ year^−1^)	+ 0.003**	+ 0.012**	+ 0.005**	+ 0.003, n.s.
TOC:TON (kg kg^−1^ year^−1^)	+ 0.309**	+ 0.276**	+ 0.157*	+ 0.171, n.s.
(e) Stream fluxes vs. deposition				
NO_3_ (stream) : TIN (dep) (year^−1^)	− 0.0003*	− 0.004**	0.000, n.s.	+ 0.001, n.s.
TN (stream) : TIN (dep) (year^−1^)	+ 0.006**	+ 0.004*	+ 0.004**	+ 0.003, n.s.

^a^1993–2017 at Øygardsbekken

All stations, apart from Øygardsbekken (NILU station Vikedal), have shown a significant increase in rainfall. The deposition of NO_3_ shows a downward trend at all stations except Langtjern (NILU station Brekkebygda). Although there was a significant decrease in the concentrations of NO_3_ and NH_4_ in precipitation at this station, increased rainfall counteracted this effect and kept the total deposition relatively stable over time. Only Storgama (NILU station Treungen) showed a significant decrease in NH_4_ deposition. If one considers TIN (the sum of NO_3_ and NH_4_) there has been a significant decrease at all stations, except at Langtjern (Fig. 2b, Table 3).

#### Streams

All sites except Øygardsbekken showed significant downward trends in annual flow-weighted concentrations of NO_3_ (Table 3). At Storgama, the strong decrease in streamwater NO_3_ concentrations also resulted in a significant reduction in TN concentrations despite that both TOC and TON increased during the same period. All sites showed upward trends in TOC concentrations, whereas upward trends in TON concentrations were only detected at Storgama and Birkenes.

**Fig. 3:**
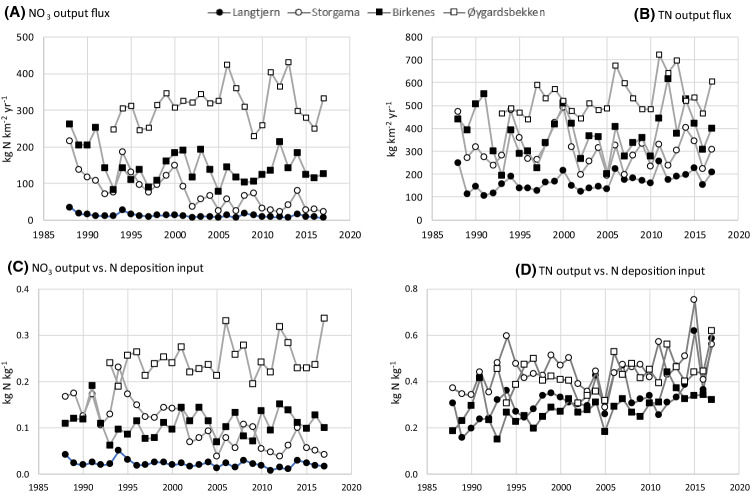
Stream water fluxes; **a** NO_3_, **b** TN, **c** NO_3_ output vs. atmospheric N input, **d** TN output vs. atmospheric N input

Two sites, Langtjern and Øygardsbekken, showed a significant increase in water flow (Table 3). There was a significant decrease in NO_3_ fluxes at the two easternmost catchments, but not at Birkenes and Øygardsbekken (Fig. 3a). All sites exhibited upward trends in fluxes of TOC, and apart from Birkenes, also in TON. Only Langtjern experienced a significant increase in TN fluxes during the study period (Fig. 3b). All sites, except for Øygardsbekken, showed a significant decrease in NO_3_ : TN ratios, and increases in TON : TN and TOC : TON ratios (Table 3).

When comparing output fluxes in the streams with inputs from atmospheric deposition—i.e., considering outputs as a fraction of TIN inputs—there has been reduced NO_3_ leaching at Langtjern and Storgama (Table 3, Fig. 3c). Birkenes and Øygardsbekken showed no signs of decreased leaching. There has been an increase in TN leaching at all sites except at Øygardsbekken (Table 3, Fig. 3d).

### Temporal and spatial progression of N losses vs. atmospheric inputs

**Fig. 4:**
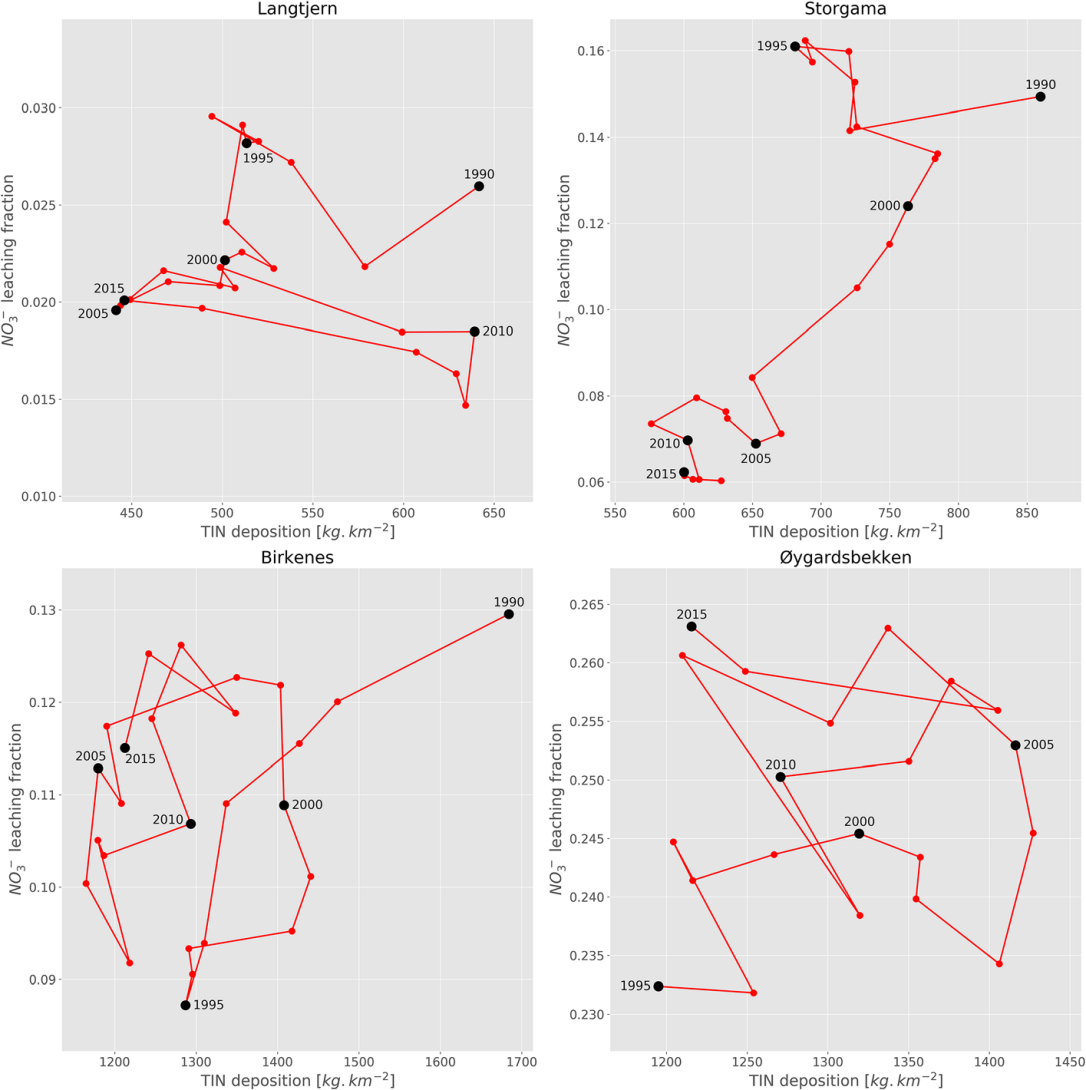
Trajectories showing five-year running means for NO_3_ leaching (fraction of atmospheric N deposition lost in stream water) vs. TIN deposition at the study sites. Note different scales in the figure panels

The long-term data illustrate that there is no simple relationship between atmospheric inputs and catchment N leaching losses on an annual basis. Even when aggregating to five-year means the variation is considerable—probably as a result of large year-to-year variations in the seasonal climate (Fig. 4). Examples are the temporary increase in N deposition levels at Langtjern around 2010 and at Storgama and Birkenes around 2000. Despite this temporal variation, some site-specific patterns emerge when data are aggregated to five-year running means.

The five-year means from 1990 to 2015 at Langtjern, Storgama, and Birkenes show about 30% lower N deposition levels and less NO_3_ leaching today (2015) compared to the early period (1990) (Fig. 4) suggesting that reductions in N deposition have led to lower leaching of NO_3_. The relative reductions in NO_3_ leaching during the period were 11% at Birkenes, 22% at Langtjern, and 58% at Storgama. At Øygardsbekken, NO_3_ leaching has increased (relative change: 13%) despite that the N deposition level around 2015 was about the same as in the early period (1995). It is also interesting to note that the reductions in NO_3_ leaching seem to stagnate (or converge) at Langtjern and Storgama after 2005, and that NO_3_ leaching at Birkenes has increased somewhat after 1995.

### Comparison with N trends in larger rivers and lakes in the same region

**Fig. 5:**
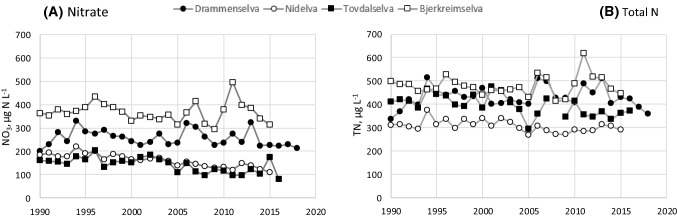
Annual mean NO_3_ and TN concentrations in larger rivers where the study catchments are located; Langtjern in River Drammenselva, Storgama in River Nidelva, Birkenes in River Tovdalselva, and Øygardsbekken in River Bjerkreimselva

**Fig. 6:**
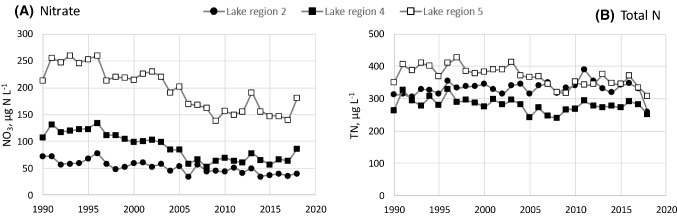
Concentrations of NO_3_ and TN in autumn samples from lakes in regions where the study catchments are located. Langtjern and Storgama are located in region 2 (15 lakes), Birkenes in region 4 (14 lakes), and Øygardsbekken in region 5 (11 lakes)

The decreasing NO_3_ trends in the easternmost catchments and absence of a clear trend in Øygardsbekken are consistent with long-term data from large rivers (Kaste et al. 2018; Norwegian Environment Agency 2018) and small lakes (Garmo and Skancke 2019) covering the same gradient in southern Norway (Figs. 5a, 6a). Apart from River Bjerkreimselva (where Øygardsbekken is located), the time series from rivers and lakes show reduced NO_3_ concentrations at most sites since 1990—which is consistent with the decrease in N deposition. In contrast to NO_3_, TN has remained relatively unchanged in the same rivers and lakes during the past 25 years (Figs. 5b, 6b).

## Discussion

### Catchment responses to reductions in N deposition

Many upland sites that received high N deposition loads during the 1970s and 1980s (Schöpp et al. 2003) have experienced significantly reduced NO_3_ concentrations in surface waters during the last 10–20 years (Kopacek et al. 2005; Vuorenmaa et al. 2018). These observations are in line with results from the European NITREX project (Tietema et al. 1998) and the Norwegian RAIN project (Wright et al. 1993), where nitrogen deposition was experimentally excluded by roof structures at several small forested sites to study ecosystem responses to changes in N deposition loads. Decreased N input at the high-deposition sites resulted in rapid and large reduction in NO_3_ concentrations in drainage water.

The four sites in Norway studied here have much lower levels of N deposition. The 30-year data record suggest that at three of four sites, decreased N deposition caused decreased loss of NO_3_ in streamwater. At Øygardsbekken NO_3_ fluxes in streamwater showed no significant change. The easternmost catchments, Langtjern and Storgama, have the highest N retention and have also shown the largest relative reductions in N leaching. Although TIN deposition is significantly reduced also in southernmost and southwestern Norway since 1988, the deposition levels at Øygardsbekken and Birkenes remain 2 to 3 times higher than at Storgama and Langtjern, respectively (Fig. 2). The east–west gradient in N deposition levels may partly explain the slower reduction in NO_3_ leaching at Birkenes and Øygardsbekken compared with the easternmost catchments. However, the lower NO_3_ leaching rate at Birkenes compared to Øygardsbekken leaves the open question why the NO_3_ retention capacity vary that much among catchments that receives approximately the same amount of N from atmospheric sources. Therefore, the differences observed in stream responses cannot be explained by the N deposition level alone, but probably related to different catchment characteristics such as bedrock type, soil properties, vegetation cover, slope, climatic factors, or legacies from former pollution history.

### Influence of catchment characteristics

The most pronounced differences between the catchments are related to the land cover (Table 1). 90% of the Birkenes catchment is covered by productive forest, while Langtjern has a large proportion (~ 70%) of low-productive forest. Storgama has only scattered trees on shallow soils and a large proportion of exposed bedrock, while Øygardsbekken is dominated by an open, non-forested, landscape with shrubs on shallow soils and large areas with exposed bedrock. The large proportion of non-forested areas with shallow soils or exposed bedrock at Storgama and Øygardsbekken, together with high hydrological flushing rates, probably reduces the catchment’s capacity to retain N that is added from atmospheric sources (Kaste et al. 1997). It is, therefore, somewhat surprising that Storgama showed the fastest response to reduced N deposition in terms of reduced NO_3_ leaching. Possible explanations might be milder winters, longer growing season allowing increased uptake and build-up of organic matter, and increased precipitation that might have stimulated denitrification in patches with peaty soils (Anderson et al. 2015). Storgama has, together with Langtjern, the largest percentage of peatlands (22 and 16%, respectively) and the relative importance of denitrification might have increased in both catchments as N deposition has declined and the climate has become wetter. In Birkenes, the slight increase in NO_3_ leaching after 1995 might be related to slower growth and reduced N uptake in an ageing (135-year old) forest.

### Role of climatic factors

The sites are located along a relatively large gradient in annual mean precipitation,^1^ from 791 mm year^−1^ at Langtjern to 2063 mm year^−1^ at Øygardsbekken (Table 1). Besides increasing the scavenging and wash-out of pollutants from the air, high precipitation also increases the risk for N losses during hydrological flushing events. With its relatively steep slopes (difference between highest and lowest point = 360 m) on thin soils Øygardsbekken is especially vulnerable to N leaching losses.

Future climate change will pull in different directions with respect to N leaching: Deposition may increase, due to increasing rainfall and wash-out of pollutants from air (Hole and Engardt 2008), higher temperature and longer growing season will result in greater plant uptake and potentially less N leakage. On the other hand, more NO_3_ may leak as a result of hydrological episodes (runoff after heavy rain) and increased decomposition and mineralisation of organic matter. Results from the present study indicate that decreasing N deposition has led to reduced NO_3_ leaching at the two easternmost catchments (Langtjern and Storgama) that have the lowest annual precipitation, the lowest annual temperature, relatively stable winter climate and the lowest N deposition.

An increasing trend in air temperature during the past 25 years may have contributed to reduced N leaching by a prolonged growing season with increased potential for N uptake by plants. On the other hand, milder winter climate with reduced snowpack may result in either decreased or increased catchment N leaching depending on interactions with N deposition, soil temperature regime and winter discharge. de Wit et al. (2008) found that at Langtjern and Storgama, milder winter climate resulted in decreased winter and spring concentrations and fluxes of NO_3_. At Birkenes and Øygardsbekken, milder and more rainy winters with frequent flood episodes might have increased the N losses during the dormant season.

### Interaction with organic matter

In contrast to NO_3_, concentrations and fluxes of TON have increased at all sites over the past 30 years (Table 3). This is almost certainly due to the increased fluxes of DOM, measured as TOC, a phenomenon observed over large parts of western Europe and eastern North America (Monteith et al. 2007). The increase in TOC is ascribed to declining trends in S deposition and sea-salts. Lower ionic strength in soil water caused by the decline in SO_4_ and sea-salts increases the solubility and mobility of organic compounds (de Wit et al. 2007). In addition, climate change with increasing and more intense rainfall together with increased vegetation growth has led to greater leaching of organic matter from the soil (de Wit et al. 2016; Finstad et al. 2016). In southern Norway, there is a strong regional gradient in TOC, with the highest concentrations in the east and lowest in the western parts of the country (Larsen et al. 2011).

The TOC:TON ratio in streamwater at the four sites appears to reflect the C:N ratio in the organic soil layers in the catchment. Øygardsbekken has the lowest TOC:TON ratio in streamwater (18) and also low C:N ratio in soil (mean: 23; Sjøeng et al. 2007), while Langtjern has the highest TOC:TON ratio in streamwater (45) and the highest C:N ratio in soil (mean: 41; SFT 2001).

At all four sites the loss of TN in streamwater constituted 30–50% of the deposition of N. Losses of gaseous N via denitrification represent an uncertainty in catchment N budgets, but fluxes at the study sites are assumed to be generally low, as a major fraction of the catchments are well-drained. This implies that the study sites to varying degrees continue to accumulate N, most likely in soil organic matter. Accumulation of N in the soil organic matter pool can lead to reduced C:N ratios, or constant C:N ratio if new organic matter accumulates. At a long-term experimental N addition to a whole forested catchment at Gårdsjön, Sweden, about half of the added N went to make new organic matter and half went to lower the C:N ratio of existing organic matter (Moldan et al. 2018). Lowering of the C:N ratio in soil organic matter may in the long-term lead to “N saturation”, manifest by increased leaching of NO_3_ to streamwater. The 30-year records presented here, however, do not suggest that N saturation is occurring. However, low C:N ratios of DOM at Øygardsbekken in SW Norway suggests long-term N-enrichment of OM, which may explain why this catchment had the highest NO_3_ leaching rate and also lacked a clear response to the decline in N deposition that has occurred over the past 30 years.

## Conclusions

Even though the N deposition levels in southern Norway, both historically and at present, are moderate compared high-deposition sites in Europe, the ecosystems are highly sensitive and have a limited capacity to retain N from atmospheric sources. This is especially the case in upland catchments such as Øygardsbekken in SW Norway, where N deposition levels still are relatively high and the N storage capacity low due to thin soils, poor vegetation cover without forest and high precipitation rates with frequent flushing events through the catchment soils.

The 30-year data records examined show that at three of four sites, decreased N deposition caused decreased loss of NO_3_ to streamwater. At the fourth site, Øygardsbekken, NO_3_ fluxes in streamwater showed no significant change. In contrast to NO_3_, flow-weighted concentrations of TON increased at all four sites during the same period.

Given that TN losses to streamwater only constituted 30–50% of the atmospheric N deposition, the study sites seem to continue to accumulate N, presumably in soil organic matter. Losses via denitrification are unknown but assumed to be low, as a major fraction of the catchments are well-drained.

Low C:N ratios of DOM at Øygardsbekken suggests long-term N-enrichment of soil organic matter, which may explain why this catchment had the highest NO_3_ leaching rates and lacked a clear response to the decline in N deposition during the preceding 30 years.

Although the N deposition has been reduced in recent years, ambient loads might still exceed long-term sustainable levels in these vulnerable ecosystem types. It is therefore uncertain whether the N leaching in catchments such as Øygardsbekken will decrease from today’s level without further reductions in atmospheric N inputs.

## Footnotes

1 30-year mean 1961–1990.
